# An outcome-driven threshold for pulse pressure amplification

**DOI:** 10.1038/s41440-024-01779-4

**Published:** 2024-07-22

**Authors:** Qi-Fang Huang, De-Wei An, Lucas S. Aparicio, Yi-Bang Cheng, Fang-Fei Wei, Yu-Ling Yu, Chang-Sheng Sheng, Wen-Yi Yang, Teemu J. Niiranen, José Boggia, Katarzyna Stolarz-Skrzypek, Valérie Tikhonoff, Natasza Gilis-Malinowska, Wiktoria Wojciechowska, Edoardo Casiglia, Krzysztof Narkiewicz, Jan Filipovský, Kalina Kawecka-Jaszcz, Tim S. Nawrot, Ji-Guang Wang, Yan Li, Jan A. Staessen, The International Database of Central Arterial Properties for Risk Stratification (IDCARS) Investigators, Lucas S. Aparicio, Jessica Barochiner, Blerim Mujaj, Lutgarde Thijs, Jan A. Staessen, Fang-Fei Wei, Wen-Yi Yang, Zhen-Yu Zhang, De-Wei An, Yi-Bang Cheng, Qian-Hui Guo, Jian-Feng Huang, Qi-Fang Huang, Yuan-Yuan Kang, Yan Li, Chang-Yuan Liu, Chang-Sheng Sheng, Ji-Guang Wang, Ying Wang, Dong-Yan Zhang, Wei Zhang, Jan Filipovský, Jitka Seidlerová, Eeva P. Juhanoja, Antti M. Jula, Annika S. Lindroos, Teemu J. Niiranen, Sam S. Sivén, Edoardo Casiglia, Alessandra Pizziol, Valérie Tikhonoff, Babangida S. Chori, Benjamin Danladi, Augustine N. Odili, Henry Oshaju, Wiesława Kucharska, Katarzyna Kunicka, Natasza Gilis-Malinowska, Krzysztof Narkiewicz, Wojciech Sakiewicz, Ewa Swierblewska, Kalina Kawecka-Jaszcz, Katarzyna Stolarz-Skrzypek, Catharina M. C. Mels, Ruan Kruger, Gontse G. Mokwatsi, Aletta E. Schutte, Gavin R. Norton, Angela Woodiwiss, Daniel Ackermann, Murielle Bochud, Georg Ehret, Ramón Álvarez-Vaz, Anna C. Rios, Florencia Carusso, Mariana Sottolano, José Boggia, Luciana Borgarello, Sebastián Robaina, Paula Moliterno, Oscar Noboa, Alicia Olascoaga, Alicia da Rosa, Nadia Krul, Matias Pécora

**Affiliations:** 1 Department of Cardiovascular Medicine, Shanghai Key Laboratory of Hypertension, Shanghai Institute of Hypertension, State Key Laboratory of Medical Genomics, National Research Center for Translational Medicine, Ruijin Hospital, Shanghai Jiaotong University School of Medicine, Shanghai, China; 2 Non-Profit Research Association Alliance for the Promotion of Preventive Medicine, Mechelen, Belgium; 3 Research Unit Environment and Health, Department of Public Health and Primary Care, University of Leuven, Leuven, Belgium; 4 Servicio de Clínica Médica, Sección Hipertensión Arterial, Hospital Italiano de Buenos Aires, Buenos Aires, Argentina; 5 Department of Cardiology, The First Affiliated Hospital of Sun Yat-Sen University, Guangzhou, Guangdong China; 6 Department of Cardiology, Shanghai General Hospital, Shanghai, China; 7 Department of Chronic Disease Prevention, Department of Medicine, Turku University Hospital and University of Turku, Turku, Finland; 8 Centro de Nefrología and Departamento de Fisiopatología, Hospital de Clínicas, Universidad de la República, Montevideo, Uruguay; 9 First Department of Cardiology, Interventional Electrocardiology and Hypertension, Jagiellonian University Medical College, Kraków, Poland; 10 Department of Medicine, University of Padua, Padua, Italy; 11 Hypertension Unit, Department of Hypertension and Diabetology, Medical University of Gdańsk, Gdańsk, Poland; 12 Faculty of Medicine, Charles University, Pilsen, Czech Republic; 13 Biomedical Sciences Group, Faculty of Medicine, University of Leuven, Leuven, Belgium; 14 Buenos Aires, Argentina; 15 Noordkempen, Belgium; 16 Jing Ning, China; 17 Pilsen, Czech Republic; 18 Finn-Home, Finland; 19 Padova, Italy; 20 Abuja, Nigeria; 21 Gdańsk, Poland; 22 Kraków, Poland; 23 Potchefstroom, South Africa; 24 Johannesburg, South Africa; 25 Bern, Geneva and Lausanne Switzerland; 26 Montevideo, Uruguay

**Keywords:** Pulse pressure amplification, Waveform analysis, Cardiovascular risk, Population science

## Abstract

Pulse pressure amplification (PPA) is the brachial-to-aortic pulse pressure ratio and decreases with age and cardiovascular risk factors. This individual-participant meta-analysis of population studies aimed to define an outcome-driven threshold for PPA. Incidence rates and standardized multivariable-adjusted hazard ratios (HRs) of cardiovascular and coronary endpoints associated with PPA, as assessed by the SphygmoCor software, were evaluated in the International Database of Central Arterial Properties for Risk Stratification (*n* = 5608). Model refinement was assessed by the integrated discrimination (IDI) and net reclassification (NRI) improvement. Age ranged from 30 to 96 years (median 53.6). Over 4.1 years (median), 255 and 109 participants experienced a cardiovascular or coronary endpoint. In a randomly defined discovery subset of 3945 individuals, the rounded risk-carrying PPA thresholds converged at 1.3. The HRs for cardiovascular and coronary endpoints contrasting PPA < 1.3 vs ≥1.3 were 1.54 (95% confidence interval [CI]: 1.00–2.36) and 2.45 (CI: 1.20–5.01), respectively. Models were well calibrated, findings were replicated in the remaining 1663 individuals analyzed as test dataset, and NRI was significant for both endpoints. The HRs associating cardiovascular and coronary endpoints per PPA threshold in individuals <60 vs ≥60 years were 3.86 vs 1.19 and 6.21 vs 1.77, respectively. The proportion of high-risk women (PPA < 1.3) was higher at younger age (<60 vs ≥60 years: 67.7% vs 61.5%; *P* < 0.001). In conclusion, over and beyond common risk factors, a brachial-to-central PP ratio of <1.3 is a forerunner of cardiovascular coronary complications and is an underestimated risk factor in women aged 30–60 years. Our study supports pulse wave analysis for risk stratification.

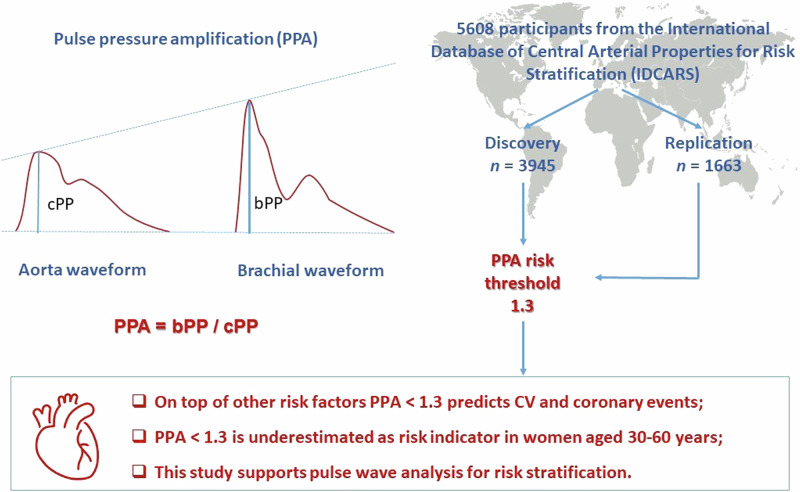

## Introduction

Pulse pressure (PP), the difference between systolic and diastolic blood pressure (BP), oscillates around mean arterial pressure and increases from the central to the peripheral arteries, causing PP amplification (PPA) [1–5]. PPA is most commonly represented by the ratio of brachial-to-aortic PP [1–5]. Over the human lifespan, PPA decreases as a consequence of arterial stiffening associated with aging and further promoted by hypertension, dyslipidemia, diabetes, and chronic kidney disease [4, 6]. Stiffening of the central elastic arteries leads to the earlier return of backward waves from peripheral reflection sites [1, 5], thereby increasing central systolic pressure, so that the ratio of brachial-to-aortic PP decreases. Among healthy, normotensive individuals enrolled in the Anglo-Cardiff Collaborative Trial (ACCT), PPA decreased from 1.72 in teenagers to 1.25 in octogenarians [7]. In populations [2] and patients [8], arterial stiffening, as captured by lower PPA, is associated with higher cardiovascular risk [2, 3], coronary artery diseases [9], deterioration of renal function [8], and mortality [3]. However, few prospective studies focused on the incidence of major cardiovascular complications in relation to PPA. To the best of our knowledge, there is therefore no published outcome-driven PPA threshold, which might alert clinicians for the risk of cardiovascular complications related to falling PPA. To address this issue, the current individual-participant level meta-analysis defined, calibrated and validated an outcome-driven threshold for PPA, using the International Database of Central Arterial Properties for Risk Stratification (IDCARS) [10].

## Methods

### Study participants

The study protocol paper describes the construction of the IDCARS database [10]. The longitudinal studies extracted from the IDCARS resource qualified for the current analysis, if information on brachial and central BP and cardiovascular risk factors was available at baseline, if the arterial pulse waveform had been measured, if follow-up included both fatal and nonfatal endpoints, if study reports were published in peer-reviewed journals, and if the study participants were representative for a population. All studies complied with the Helsinki Declaration on research in humans [11] and were approved by the competent Institutional Review Boards. Participants provided written informed consent. All data were stripped from personal identifiers, and if required by national legislations, additional ethical clearance was obtained. Supplementary Table S1 lists cohort-specific information on the catchment area, sampling strategies, timeframes of recruitment and follow-up, and initial participation rates. Enrolment took place from 1985 until 2015. For the present analysis, baseline refers to the first measurement of central and peripheral BP along with cardiovascular risk factors (October 2000 until February 2016). Across studies, the last follow-up took place from October 2012 to December 2018 (Supplementary Table S1). Of 6650 IDCARS participants qualifying for analysis, we excluded 1042 because they were younger than 30 years without endpoints (*n* = 954), peripheral PP was >130 mmHg (*n* = 10), central systolic BP was <70 mmHg (*n* = 1) or >230 mmHg (*n* = 1), central diastolic BP was >150 mmHg (*n* = 1) or <55 mmHg (*n* = 15), or because the pulse wave analysis was missing (*n* = 60). This left 5608 participants for statistical analysis.

### Pulse wave analysis

Brachial BP, measured immediately prior to the hemodynamic assessment after participants had rested for 5 up to 15 min in the supine position, was the average of two consecutive readings. Experienced observers recorded the radial arterial waveform at the dominant arm during an 8-s period by applanation tonometry. They used a high-fidelity SPC-301 micromanometer (Millar Instruments Inc., Houston, TX), interfaced with a SphygmoCor CvMS device and a laptop computer running SphygmoCor software (AtCor Medical Inc., Itasca, IL). Recordings were discarded, if systolic or diastolic variability of consecutive waveforms exceeded 5% or if the amplitude of the pulse wave signal was below 80 mV, or if the operator index was <70%. From the radial signal, the SphygmoCor software reconstructs the aortic pulse wave by means of a validated generalized transfer function [12]. Estimates of central blood pressure were calibrated on brachial systolic and diastolic BP. PPA was the brachial-to-aortic PP ratio.

### Ascertainment of endpoints

We ascertained the incidence of fatal and nonfatal endpoints from the appropriate sources in each country. The primary endpoint was a composite cardiovascular outcome consisting of cardiovascular mortality, sudden death and nonfatal endpoints, including myocardial infarction, heart failure, stroke, and coronary arterial revascularization. The coronary endpoint comprised sudden death and fatal and nonfatal myocardial infarction and coronary revascularization. The endpoints are listed in Supplementary Table S2. All endpoints were validated against hospital files or medical records held by primary care physicians or specialists. Only the first event within each category was considered in the analysis of outcome.

### Statistical analysis

Statistical methods are described in detail in the online-only Data Supplement (pp. 2–5). In exploratory analyses, incidence rates of endpoints were tabulated by tertiles of the PPA distribution, while applying the direct method for standardizing rates for cohort, sex and age (<40, 40–59, ≥60 years). The cumulative incidence of the cardiovascular and coronary endpoints was plotted, while accounting for cohort, sex, age and heart rate.

Multivariable-adjusted Cox models accounted for cohort (random effect), sex, age, heart rate, body mass index, smoking and drinking, the total-to-HDL serum cholesterol ratio, the glomerular filtration rate estimated from serum creatinine by the chronic kidney disease epidemiology collaboration equation [13], antihypertensive drug intake, history of cardiovascular disease and diabetes. The proportional hazards assumption was checked by the Kolmogorov-type supremum test.

After stratification for sex, median age (53.6 years) and cohorts (*n* = 9; Supplementary Table S1), a random function was applied to subdivide the total IDCARS study population (*n* = 5608) into a discovery (*n* = 3945) and replication (*n* = 1663) sample. The size of the discovery dataset required 171 cardiovascular endpoints to demonstrate a difference between high-risk (above PPA risk threshold) and low-risk (below PPA risk thresholds) individuals with the α-level and power set at 0.05 and 0.80, respectively [14]. The default significance throughout the current study was a two-tailed α-level of ≤0.05 with the SE to compute two-sided confidence intervals [CI] set at 1.96. However, given the prior probability in the discovery dataset, in the replication analysis, the α-level was a one-tailed level of ≤0.05 and the SE to compute CIs was 1.65.

To determine an operational threshold for PPA in the discovery dataset, a two-pronged strategy [15, 16] was applied using Cox regression. First, multivariable-adjusted HRs were computed for 0.1 increments in PPA from the 10th to the 90th percentile of the PPA distribution. These HRs expressed the risk in participants, whose PPA exceeded the cut-off point vs the average risk in the whole population. The HRs with CIs were plotted as function of increasing PPA thresholds to assess at which PPA level the upper 95% confidence limit of the HRs crossed unity, signifying decreased risk [15]. Next, PPA thresholds were obtained by determining the PPA levels yielding a 5-year risk equivalent to the risk associated with a brachial systolic BP of 120-, 130-, 140- and 160 mmHg [16]. Model calibration was evaluated by comparing the predicted risk against overoptimism-corrected Kaplan–Meier estimates in PPA quintiles. The performance of PPA in risk stratification was assessed from 2-by-2 tables providing specificity, sensitivity and related statistics, the area (AUC) under the receiver operating curve (ROC), the area (AUC) under the receiver operating curve, and by the integrated discrimination improvement (IDI) and the net reclassification improvement (NRI) [17].

Finally, subgroup analyses were conducted in participants stratified by sex, age (<60 vs ≥60 years), median systolic BP (<130 vs ≥130 mmHg) and antihypertensive treatment status. To compare relative risk across these strata, deviation from mean coding [18] was applied. In a further sensitivity analysis, models for the coronary endpoint were additionally adjusted for diastolic BP, the driving force of the coronary circulation.

## Results

### Baseline characteristics of participants

Table 1 lists the main characteristics of the 5608 analyzed participants. Mean age at baseline was 54.2 years. Among all participants, 2988 (53.3%) had hypertension, 1946 (34.7%) were on antihypertensive treatment. Of 1946 patients reporting information on antihypertensive drugs, 672 (34.5%) and 1274 (65.5%) were taking a single agent or combination therapy, respectively. Drug classes taken were diuretics in 648 (33.3%) patients, β-blockers in 747 (38.4%), inhibitors of the renin–angiotensin system in 1184 (60.8%), and vasodilators in 833 (42.8%). Among the 5608 study participants (Table 1), 3035 (54.1%) were women. The PPA distributions is shown in Supplementary Fig. S1. The association between PPA and age is shown in Supplementary Fig. S2. Compared to women, more men reported smoking cigarettes (33.7% vs 10.2%) or consuming alcoholic beverages (75.4% vs 28.9%).

**Table 1:** Baseline characteristics of participants

Characteristic	Discovery	Replication	All
Number in group	3945	1663	5608
Number with characteristic (%)			
Ethnicity			
White Europeans	1684 (42.7)	701 (42.2)	2385 (42.5)
Chinese	1279 (32.4)	544 (32.7)	1823 (32.5)
South Americans	982 (24.9)	418 (25.1)	1400 (25.0)
Women	2134 (54.1)	901 (54.2)	3035 (54.1)
Hypertension	2076 (52.6)	912 (54.8)	2988 (53.3)
Treated hypertension	1346 (34.1)	600 (36.1)	1946 (34.7)
Diabetes mellitus	246 (6.2)	93 (5.6)	339 (6.0)
History of cardiovascular disease	555 (14.1)	238 (14.3)	793 (14.1)
Current smoking	853 (21.6)	326 (19.6)	1179 (21.0)
Drinking alcohol	1990 (50.4)	826 (49.7)	2816 (50.2)
Mean of characteristic (SD)			
Age, y	54.2 (14.5)	54.2 (14.1)	54.2 (14.4)
Body mass index, kg/m^2^	25.8 (4.9)	25.9 (4.8)	25.8 (4.8)
Brachial blood pressure, mmHg			
Systolic	134.1 (20.9)	134.1 (21.2)	134.1 (21.0)
Diastolic	80.0 (10.6)	80.5 (10.8)	80.1 (10.7)
Pulse pressure	54.1 (16.3)	53.6 (16.3)	53.9 (16.3)
Central blood pressure, mmHg			
Systolic	123.7 (21.0)	123.9 (21.5)	123.7 (21.2)
Diastolic	81.0 (10.8)	81.5 (11.0)	81.2 (10.9)
Pulse pressure	42.6 (16.0)	42.4 (16.2)	42.6 (16.1)
Heart rate, beats per min	65.5 (11.4)	65.6 (11.5)	65.5 (11.4)
Total serum cholesterol, mg/dL	194.8 (38.6)	196.8 (39.8)	195.4 (38.9)
HDL serum cholesterol, mg/dL	57.4 (15.2)	57.6 (15.2)	57.5 (15.2)
Total-to-HDL cholesterol ratio	3.6 (1.1)	3.6 (1.1)	3.6 (1.1)
Glomerular filtration rate (mL/min/1.73 m^2^)	82.4 (19.9)	82.6 (19.1)	82.5 (19.6)
Blood glucose, mg/dL	90.6 (18.8)	90.9 (20.2)	90.7 (19.2)
Pulse pressure amplification	1.31 (0.19)	1.31 (0.19)	1.26 (1.16–1.42)
Median follow-up (IQR), years	4.1 (3.6–6.9)	4.2 (3.6–7.5)	4.1 (3.6–6.9)

After stratification for sex, median age (53.6 years) and cohorts, a random function was applied to subdivide the total IDCARS study population into a discovery and replication sample, which were well matched (*P* ≥ 0.09 for all characteristics). Current smoking was inhaling tobacco smoke on a daily basis. Drinking alcohol was the occasional or daily consumption of alcoholic beverages. Diabetes mellitus was use of anti-diabetic drugs, fasting blood glucose ≥126 mg/dL, random blood glucose ≥200 mg/dL, a self-reported diagnosis, or diabetes documented in practice or hospital records. Hypertension was a brachial blood pressure of ≥140 mmHg systolic or ≥90 mmHg diastolic, or use of antihypertensive drugs. The glomerular filtration rate was derived from serum creatinine using the chronic kidney disease epidemiology collaboration formula

*HDL* high-density lipoprotein

### Incidence of endpoints

**Fig. 1:**
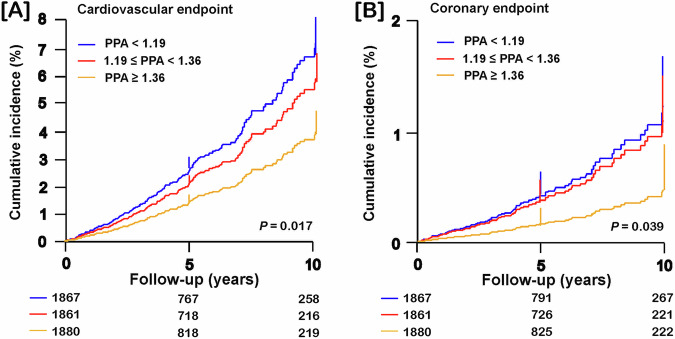
Cumulative incidence of the cardiovascular and coronary endpoints by tertiles of pulse pressure amplification. Cumulative incidence of the cardiovascular (**A**) and coronary (**B**) endpoints was derived by Cox regression with adjustment for cohort, sex and age. *P* values denote the overall significance of the difference between the PPA categories. Vertical lines denote the SE. Tabulated data are the number of participants at risk at 5-year intervals

Over a median follow-up of 4.1 years (interquartile range (IQR): 3.6–6.9 years; 5th–95th percentile interval: 2.2–12.1 years), of 5608 participants, 255 (4.55%) experienced a cardiovascular endpoint and 109 (1.94%) a coronary endpoint, resulting in rates of 9.59 events per 1000 person-years (confidence interval (CI): 8.82–10.4) and 4.05 events per 1000 person-years (CI: 3.53–4.57), respectively. Across tertiles of the PPA distribution, the crude rates of cardiovascular and coronary endpoints decreased with higher PPA category (Supplementary Table S3). Figure 1 shows the cumulative incidence of the cardiovascular and coronary endpoints by PPA tertiles with adjustment for cohort (random effect), sex and age. Given that in the whole study population, PPA was a risk factor, in the next step of the analysis, we first determined an outcome-driven threshold in the discovery sample.

### Analysis of the discovery sample

**Fig. 2:**
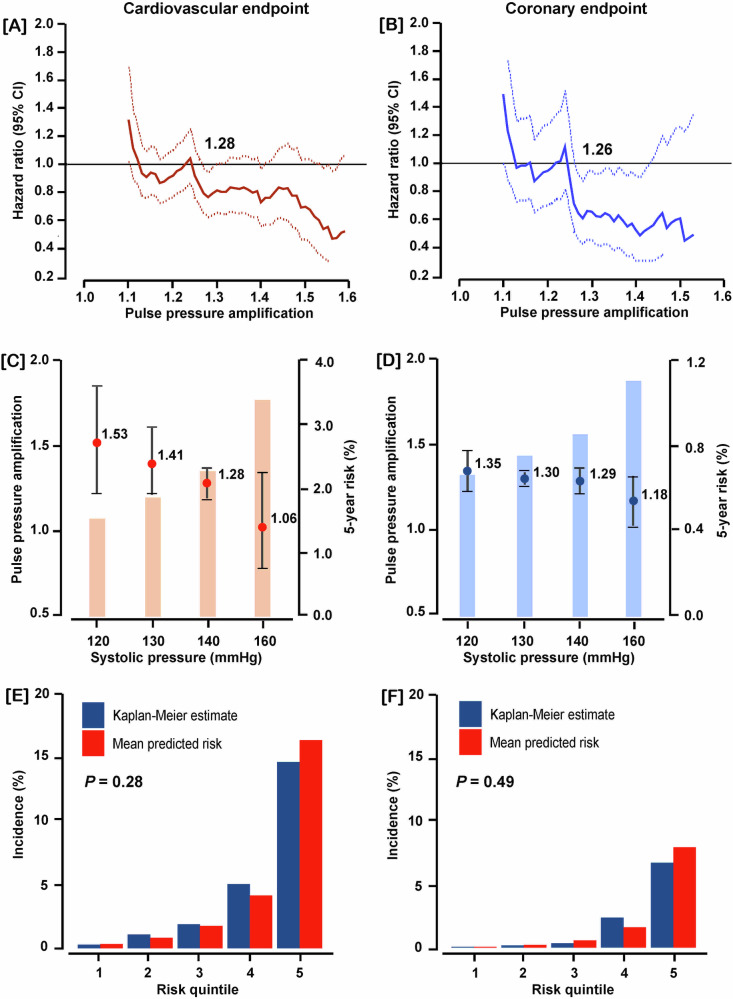
Threshold and calibration of pulse pressure amplification (PPA) in the discovery sample including 3945 participants. Hazard ratios (HRs) express the risk at each PPA level relative to the average risk in the whole discovery sample for cardiovascular (**A**) and coronary endpoints (**B**). The upper confidence limit crosses unity, denoted by the vertical line, at 1.28 and 1.26 for cardiovascular and coronary endpoints, signifying decreased risk. The PPA levels yielding equivalent 5-year risks compared with a systolic blood pressure of 140 mmHg were 1.28 and 1.29 for the cardiovascular (**C**) and coronary (**D**) endpoints, respectively. Model calibration for the cardiovascular (**E**) and coronary (**F**) endpoints demonstrated that across PPA quintiles the predicted risk was similar compared with the overoptimism-corrected Kaplan–Meier estimates. All analyses were multivariable adjusted for cohort (random effect), sex, age, body mass index, heart rate, smoking and drinking, total-to-HDL serum cholesterol ratio, estimated glomerular filtration rate, antihypertensive drug intake, history of cardiovascular disease, and diabetes

Multivariable-adjusted HRs were plotted against PPA thresholds stepwise increasing by 0.1 over the 10th–90th percentile range of the PPA distribution (Fig. 2A, B). These multivariable-adjusted HRs expressed the 5-year risks of cardiovascular and coronary endpoints associated with successively increasing PPA thresholds compared to the average risk in the discovery cohort. The upper 95% confidence limit of the HRs crossed unity at a PPA level of 1.28 and 1.26 for the cardiovascular and coronary endpoints, respectively. Furthermore, the PPA thresholds yielding a risk equivalent with a systolic BP of 140 mmHg were 1.28 (CI: 1.19–1.36) and 1.29 (CI: 1.22–1.36) for the cardiovascular and coronary endpoints (Fig. 2C, D). With rounding applied, the PPA thresholds distinguishing between low and high risk of the two endpoints endpoint were <1.3 vs ≥1.3). In the discovery dataset, the adjusted HRs contrasting PPA levels of <1.3 vs ≥1.3 were 1.54 (CI: 1.00–2.36) for the cardiovascular endpoint and 2.45 (CI: 1.20–5.01) for the coronary endpoint (Table 2). These models were well calibrated (Fig. 2E, F). A 1-SD PPA increment in the discovery cohort yielded multivariable-adjusted HRs of 0.74 (CI: 0.26–0.97) and 0.57 (CI: 0.36–0.91) for the cardiovascular and coronary endpoint (Table 2).

**Table 2:** Endpoints in relation to PPA per threshold or analyzed as continuously distributed variable

Cohort Endpoint Risk indicator	Ne/Nr	HR (95% CI)	*P* value
Discovery (*n* = 3945)			
Cardiovascular			
PPA < 1.3 vs ≥1.3	150/2230 vs 39/1715	1.54 (1.00–2.36)	0.048
PPA + 1 SD	189/3945	0.74 (0.56–0.97)	0.031
Coronary			
PPA < 1.3 vs ≥1.3	66/2230 vs 12/1715	2.45 (1.20–5.01)	0.014
PPA + 1 SD	78/3945	0.57 (0.36–0.91)	0.018
Replication (*n* = 1663)			
Cardiovascular			
PPA < 1.3 vs ≥1.3	53/949 vs 13/714	2.02 (1.07–3.78)	0.033
PPA + 1 SD	66/1663	0.61 (0.39–0.96)	0.036
Coronary			
PPA < 1.3 vs ≥1.3	25/949 vs 6/714	2.90 (1.18–7.12)	0.025
PPA + 1 SD	31/1663	0.61 (0.34–1.11)	0.085

Models were adjusted for cohort (random effect), sex, age, body mass index, heart rate, smoking and drinking, total-to-HDL serum cholesterol ratio, estimated glomerular filtration rate, antihypertensive drug intake, history of cardiovascular disease, and diabetes. Given the prior probability generated in the discovery sample, confidence interval and *P* values in the replication sample are one-sided

*Ne*/*Nr* number of events/of participants at risk

### Replication

In the replication population (Table 2), the HRs were similar, but given the prior probability generated in the discovery sample HRs are given with one-sided *P* values and confidence intervals. However, given the smaller sample size, significance was not reached for the coronary endpoint in the continuous analysis (*P* = 0.085).

### Predictive performance

Supplementary Table S4 lists the performance of the 1.3 PPA threshold in the 5-year risk stratification and the AUC for the continuously distributed PPA. For the cardiovascular and coronary endpoints, the classification estimates derived from 2-by-2 tables were broadly similar in the discovery, replication and the whole dataset with specificity ~0.40 and sensitivity ~0.80. For the continuously distributed PPA, the AUC was around 0.60.

In the discovery and replication sample and in all participants, NRI for the 1.3 PPA threshold was significant, ranging from 16.1 to 33.7% and from 30.8 to 44.9% for the cardiovascular and coronary endpoint, respectively (Table 3). The corresponding NRI estimates in the continuous analysis ranged from 9.26 to 22.7% and from 21.5 to 30.2%. For both endpoints in the per threshold as well as the continuous analyses IDI did not reach significance.

**Table 3:** Integrated discrimination improvement and net reclassification improvement for pulse pressure amplification

Cohort Endpoint Risk indicator	Integrated discrimination improvement		Net reclassification improvement	
	IDI (%)	*P*	NRI (%)	*P*
Discovery (*n* = 3945)				
Cardiovascular				
PPA < 1.3 vs ≥1.3	0.12 (−0.25, 0.50)	0.52	16.1 (1.73, 30.5)	0.028
Continuous	0.27 (−0.15, 0.69)	0.21	9.26 (−5.29, 23.8)	0.21
Coronary				
PPA < 1.3 vs ≥1.3	0.41 (−0.41,1.22)	0.33	33.0 (11.9, 54.2)	0.002
Continuous	0.40 (−0.47, 1.27)	0.36	21.6 (−0.41, 43.7)	0.054
Replication (*n* = 1663)				
Cardiovascular				
PPA < 1.3 vs ≥1.3	0.50 (−0.72, 1.71)	0.42	33.7 (10.0, 57.4)	0.005
Continuous	0.76 (−0.35, 1.88)	0.18	22.7 (−1.74, 47.1)	0.069
Coronary				
PPA < 1.3 vs ≥1.3	0.89 (−2.08, 3.85)	0.56	44.9 (11.7, 78.2)	0.008
Continuous	1.08 (−0.60, 2.76)	0.21	30.2 (−4.45, 64.8)	0.088
All (*n* = 5608)				
Cardiovascular				
PPA < 1.3 vs ≥1.3	0.16 (−0.20, 0.53)	0.38	15.2 (2.76, 27.6)	0.017
Continuous	0.29 (−0.10, 0.68)	0.15	11.8 (−0.70, 24.3)	0.064
Coronary				
PPA < 1.3 vs ≥1.3	0.49 (−0.37, 1.34)	0.26	30.8 (12.7,48.9)	0.001
Continuous	0.46 (−0.28, 1.21)	0.22	21.5 (2.85, 40.2)	0.024

The base model included cohort (random effect), sex, age, body mass index, heart rate, smoking and drinking, total-to-HDL serum cholesterol ratio, estimated glomerular filtration rate, antihypertensive drug intake, history of cardiovascular disease, and diabetes. The integrated discrimination improvement (IDI) is the difference between the discrimination slopes of the base model and the base model extended by PPA. The discrimination slope is the difference in predicted probabilities (%) between participants without and with an endpoint. The net reclassification index (NRI) is the sum of the percentages of participants reclassified correctly in individuals without and with an endpoint. IDI and NRI estimates are given with 95% confidence interval

### Subgroup and sensitivity analyses

**Fig. 3:**
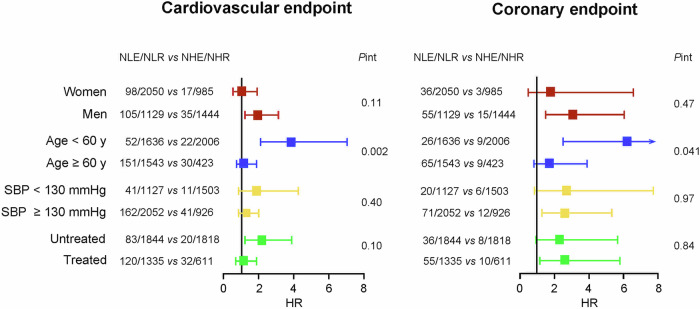
Hazard ratios expressing the risk of cardiovascular and coronary endpoints per the 1.3 pulse pressure amplification threshold by subgroups. Hazard ratios, given with 95% confidence interval accounted for cohort (random effect), sex, age, body mass index, heart rate, smoking and drinking, the total-to-HDL serum cholesterol ratio, the estimated glomerular filtration rate, antihypertensive drug intake, history of cardiovascular disease and diabetes. Adjustment for sex, age and antihypertensive treatment was omitted, if these variables defined the strata. Squares represent the point estimates of the hazard ratios (HR). Horizontal lines denote 95% confidence interval. NLE/NLR vs NHE/NHR refer to the number of participants with an event/number of participants at risk dichotomized by the threshold of pulse pressure amplification (<1.3 vs ≥1.3, respectively)

To investigate the consistency of the 1.3 PPA threshold, subgroup analyses were conducted in participants stratified by sex, age (<60 vs ≥60 years), median systolic BP (<130 vs ≥130 mmHg) and antihypertensive treatment status (Fig. 3). With full adjustments applied, the subgroup-by-PPA interaction terms were only significant if participants were categorized by age (*P* ≤ 0.041). The HRs for a cardiovascular endpoint in the age groups <60 vs ≥60 years were 3.86 (CI: 2.11–7.04) vs 1.19 (CI: 0.75–1.89); the corresponding HRs for the coronary endpoint were 6.21 (CI: 2.50–15.4) vs 1.77 (CI: 0.80–3.89). Among 2006 young (<60 years) low-risk individuals (PPA ≥ 1.3), 815 (40.6%) were women and 129 (6.43%) had isolated systolic hypertension (systolic/diastolic BP: ≥140/<90 mmHg, irrespective of treatment status), while among high-risk (PPA < 1.3) individuals in the same age band these numbers were 1107 (67.7%) and 208 (12.7%), respectively (*P* < 0.001 for both). Among 423 older (≥60 years) low-risk (PPA ≥ 1.3) participants, 172 (40.2%) were women and 128 (30.3%) had isolated systolic hypertension, while among high-risk (PPA < 1.3) individuals of similar age these numbers were 943 (61.1%) and 612 (39.7%), respectively (*P* < 0.001 for both). The proportion of women at high risk (PPA < 1.3) was higher in young individuals (<60 vs ≥60 years: 67.7% vs 61.5%; *P* < 0.001).

In multivariable models additionally adjusted for diastolic BP, the HR for coronary endpoints per threshold in the discovery dataset (PPA < 1.3 vs ≥1.3; number endpoints/individuals at risk: 66/2230 vs 12/1715) was 2.44 (CI: 1.18–5.07; *P* = 0.017) and 2.79 (CI: 1.10–7.06; *P* = 0.034) in the replication dataset (25/949 vs 6/714). The corresponding standardized HRs for PPA as continuously distributed variable were 0.57 (CI: 0.35–0.92; *P* = 0.022) and 0.64 (CI: 0.34–1.20; *P* = 0.12).

## Discussion

According to our reading of the literature, the current study breaks new grounds in two ways. First, it is a prospective population study showing that lower PPA is a risk factor for fatal combined with nonfatal cardiovascular and coronary endpoints. Foremost, it defined and replicated an outcome-driven PPA threshold of 1.3, which particularly below age 60 indicated risk of a cardiovascular or coronary endpoint.

To our knowledge, only three longitudinal population studies reported on the risk associated with PPA. The PARTAGE (Predictive Values of Blood Pressure and Arterial Stiffness in Institutionalized Very Aged Population) Study included 1126 participants (77.6% women), who were living in French and Italian nursing homes (mean age: 88.5 years) [3]. During the 2-year follow-up, 247 subjects died, and 228 experienced a major cardiovascular endpoint. A 10% PPA increase was associated with decreases in the event rates amounting to 24% and 17% for total mortality and cardiovascular events, respectively. In the current study, participants with a risk-carrying PPA had a significant higher rate of isolated systolic hypertension (25.8% vs 10.6%) and the HRs contrasting the risk of cardiovascular and coronary endpoints per PPA threshold in individuals <60 vs ≥60 years were 3.86 vs 1.19 and 6.21 vs 1.77, respectively. In the Framingham Heart Study [19], involving 2232 participants (58% women; mean age: 63 years), 151 cardiovascular endpoints occurred over 7.8 years of follow-up. Several indexes reflecting aortic stiffness were predictive, but the standardized multivariable-adjusted hazard ratio for PPA was 0.86 (CI: 0.19–3.82; *P* = 0.84). The Anglo-Cardiff Collaborative Study involved healthy people and patients with risk factors, such as hypertension, hypercholesterolemia, smoking or diabetes and patients with a history of cardiovascular disease [20]. The 10,613 participants were from 18 to 101 years old (50.8% women). In this report, PPA was expressed as the reciprocal of the traditionally used definition, so that higher values represented individuals with a relatively higher aortic pressure for a given brachial pressure. The aorta-to-brachial PP ratio averaged 0.72 in healthy subjects (1.39 according to the current PPA definition) and was consistently and significantly higher in all other groups (range of means: 0.77–0.80 [1.25–1.30 according to the current definition]) except in smokers (mean value: 0.66 [1.51]).

### Pulse pressure amplification and aging

There is large consistency among reports describing the age dependency of PPA. In both women and men, central systolic BP increased more with age than brachial systolic BP (*P* < 0.001) [21, 22]. The cross-sectional ACCT [7], including 4001 healthy, normotensive individuals, aged 18–90 years, showed that PPA decreased from 1.72 in teenagers to 1.25 in octogenarians. Another study combined volunteers recruited from the community and patients attending an open access clinic for the assessment of cardiovascular risk [23]. Patients with cardiovascular disease, diabetes or on drug treatment were excluded, but not those with hypertension. PPA decreased linearly with age (*r* = −0.70; *P* < 0.001) [23]. However, the association with age of the ratio of brachial PP to the non-augmented central PP was not statistically significant (*r* = 0.10; *P* = 0.10) [23]. In the current study, the correlation coefficient between PPA and age was −0.50 (*P* < 0.001, Supplementary Fig. S2).

### Clinical relevance

That PPA declines with aging is well established [1–5]. However, among 3642 IDCARS participants younger than 60 years, 1636 (44.9%) had a risk-carrying PPA of <1.3, indicative of early vascular aging and stiffening of the central elastic arteries. Of these high-risk individuals, 1107 (67.7%) were women. Identifications of these individuals at risk is clinically highly relevant, so that cardiovascular risk factors can be timely managed to prevention cardiovascular and coronary complications.

### Study limitations

Whilst the IDCARS database is a powerful resource, our study must be interpreted within the context of its potential limitations. First, the reconstruction of the aortic pulse wave from the radial pulse wave using the SphygmoCor technology requires the application of a generalized transfer function, which has been validated [24], but which has also been criticized [25]. For example, the SPC-301 micromanometer interfaced with the SphygmoCor device uses a single pressure sensor for applanation tonometry, but some validation studies of the generalized transfer function have utilized a servo-controlled automated tonometric system based on an arrayed sensor to avoid issues related to a manually operated single sensor [26, 27]. Second, the demographic characteristics, the period of recruitment, and the assessment of endpoint data differed between cohorts. However, the present analyses were adjusted for cohort as a random effect. By design participant-level meta-analyses allow applying the same statistical methods to all contributing cohorts, which is a strong point compared with meta-analyses of summary statistics [28]. Finally, over a median follow-up only 109 coronary endpoints occurred, probably because the low event rate among Chinese (0.49%) vs Europeans (2.64%) and South Americans (2.64%).

## Conclusions

Over and beyond common risk factors, low PPA defined as a brachial-to-central PP ratio of <1.3 is a forerunner of cardiovascular and coronary complications and is an underestimated risk factor in women aged from 30 to 60 years. Our study therefore adds to the growing evidence supporting pulse wave analysis for risk stratification in clinical centers where the technology is readily available. If the technology is unavailable, such as for instance in low- and middle-income countries, isolated systolic hypertension can be used as a proxy for PPA [29], in particular in middle-aged and older individuals [30]. However, an important caveat pertains to adolescents and young adults with isolated systolic hypertension. The risk of a cardiovascular events in such patients is similar to that of age-matched individuals without isolated systolic hypertension [31] and lower than those with combined systolic-diastolic hypertension or isolated diastolic hypertension [32, 33]. Indeed, young adults with isolated systolic hypertension appear to comprise a heterogeneous patients group, including those with increased stroke volume, normal central blood pressure but exaggerated blood pressure amplification from the central to the peripheral arteries, and those with accelerated vascular aging and premature arterial stiffness [34]. In these young patients, assessing arterial stiffness is even more indicated [35] as well as out-of-office blood pressure monitoring to exclude white-coat hypertension [36].

## Supplementary information

Supplementary Information

Supplementary Table
