# Supplementary material for: An outcome-driven threshold for pulse pressure amplification
Source: Hypertens Res. 2024 Jul 22;47(9):2478–88. doi: 10.1038/s41440-024-01779-4 (PMC11374666; doi:10.1038/s41440-024-01779-4)
Supplement: Supplementary file 1 — Supplementary Information [file 41440_2024_1779_MOESM1_ESM.doc]

***Hypertension Research***

***Data Supplement***

This Data Supplement formed part of the original submission and has been peer reviewed.
Supplement to: “*An outcome-driven threshold for pulse pressure amplification*”.

Huang QF, An DW, Aparicio LS, Cheng YB, Wei FF, Yu YL, Sheng CS, Yang WY, Niiranen TJ, Boggia J, Stolarz-Skrzypek K, Tikhonoff V, Gilis-Malinowska N, Wojciechowska W, Casiglia E, Narkiewicz K, Filipovský J, Kawecka-Jaszcz K, Nawrot TS, Wang JG, Li Y, Staessen JA, the International Database of Central Arterial Properties for Risk Stratification (IDCARS) Investigators

**Table of contents**

**Page**

**Expanded statistical methods** S2

**Supplementary Table S1** Methods of recruitment and follow-up of the IDCARS cohorts S6

**Supplementary Table S2** Number of endpoints in 5608 participants S7

**Supplementary Table S3** Cardiovascular and coronary endpoint rates by PPA tertiles in 5608 participants S8

**Supplementary Table S4** Discriminative performance of pulse pressure amplification S9

**Supplementary Fig. S1** Distribution of pulse wave amplification in 5608 IDCARS participants. S10

**Supplementary Fig. S2** Association between pulse pressure amplification and age in 5608 IDCARS participants S11

**Expanded statistical methods**

For database management and statistical analysis, SAS software, version 9.4 (maintenance level 5) was used. The central tendency and spread of continuously distributed variables were presented as mean and standard deviation (SD). Means and proportions were compared between groups by the large sample z‑test or ANOVA for continuously distributed variables, and by Fisher exact test for categorical variables. After stratification for the 9 cohorts and sex, missing values of serum total and high-density lipoprotein (HDL) cholesterol and blood glucose were interpolated from the cohort- and sex-specific regression slopes on age. In participants with unknown status of smoking or drinking status, the indicator (dummy) variable was set to the cohort- sex- and age-specific mean of the codes (0, 1). For the cohort recruited in Buenos Aires, Argentina, alcohol consumption was extrapolated from national statistics stratified by sex and age.^1^ Unless stated otherwise, statistical significance was a 2‑sided probability of 0.05.

In exploratory analyses, incidence rates of endpoints expressed as events per 1000 person-years were tabulated by tertiles of the pulse pressure amplification (PPA) distribution, while applying the direct method for standardizing rates for cohort (*n* = 9), sex and age (<40, 40-59, ≥60 years). The 95% confidence intervals (CI) of rates were computed as R±1.96×√(R×[100-R]/*T*), where *R* is the rate and *T* is the number of participants at risk of developing an adverse outcome. Next, the cumulative incidence of the cardiovascular and coronary endpoint was plotted, while accounting for cohort, sex, age.

After stratification for sex, median age (53.6 years) and cohorts (n = 9; **Supplementary Table S1**), a random function was applied to subdivide the total IDCARS study population (*n* = 5608) into a discovery (*n* = 3945) and replication (*n* = 1663) dataset. To determine an operational threshold for PPA in the discovery sample, a two-pronged strategy^2,3^ was applied using Cox regression. First, multivariable-adjusted HRs were computed for 0.1 increments in the PPA from the 10th to the 90th percentile of the PPA ratio distribution. These HRs expressed the risk in participants, whose PPA exceeded the cut-off point *vs* the average risk in the whole population. The HRs with CI were plotted as function of increasing PPA thresholds to assess at which PPA level the lower CI of the HRs crossed unity, signifying increased risk.^2^ Next, PPA thresholds were obtained by determining the PPA levels yielding a 5‑year risk equivalent to the risk associated with an office systolic BP of 120-, 130-, 140- and 160 mm Hg.^3^ This approach involved 5 steps. In the first step, the 5‑year incidence rates of the co-primary endpoints associated with the office systolic blood pressure thresholds were computed. The second step involved determining the 5‑year incidence rates of these endpoints for PPA values increasing from the from the 10th to the 90th percentile of the PPA distribution, using steps of 0.1. In the third step, the PPA values were selected that were associated with similar 5-year risks as the BP thresholds. Next, the bootstrap distribution of the so-obtained PPA thresholds was generated by randomly resampling the study population 1000 times with replacement, using the PROC SURVEY SELECT procedure, as implemented in the SAS package. For each new sample, the first 3 steps were repeated. The fourth step accounted for tied event times, caused by resampling with replacement, by the TIES=EXACT option in the PROC PHREG procedure. Finally, the bootstrap point estimates and 95% CIs of the PPA thresholds were set at the mean ± 1.96 SEs of the bootstrap distribution. The Cox models in these analyses accounted for cohort (random effect), sex, age, heart rate, body mass index, smoking and drinking, the total-to-HDL serum cholesterol ratio, the glomerular filtration rate estimated from serum creatinine by the Chronic Kidney Disease Epidemiology Collaboration equation,^4^ antihypertensive drug intake, history of cardiovascular disease and diabetes. In these Cox models, the European IDCARS participants were pooled as a single cohort. The proportional hazards assumption was checked by the Kolmogorov-type supremum test. In Cox models including PPA as independent continuously distributed variable, hazard ratios (HRs) were expressed per 1‑SD increment. Model calibration was evaluated by comparing the predicted risk against overoptimism-corrected Kaplan-Meier estimates in PPA quintiles.

The performance of PPA in risk stratification was assessed from 2‑by‑2 tables providing specificity, sensitivity and related statistics, the area (AUC) under the receiver operating curve (ROC), the area (AUC) under the receiver operating curve, and by the integrated discrimination improvement (IDI) and the net reclassification improvement (NRI).^5^ IDI is the difference between the discrimination slopes of the basic model and the basic model extended with the PPA threshold. The discrimination slope is the difference in predicted probabilities (%) between participants with and without endpoint. The calculation of NRI^5^ involves predicting in each participant the 5‑year risk of an event from a Cox model with and without the PPA threshold. If P(up/event) is the percentage of subjects with events whose predicted probability is increased by adding the PPA threshold to the model and if P(up/nonevent) is the percentage of participants without events whose predicted probability is increased, then NRI equals 2 × [P(up/event) – P(up/nonevent)]. Finally, subgroup analyses were conducted in participants stratified by sex, age (<60 *vs* ≥60 years), median systolic BP (<130 *vs* ≥130 mm Hg) and antihypertensive treatment status. To compare relative risk across strata, deviation from mean coding^6^ was applied. This approach avoids defining an arbitrary reference group and generates confidence intervals (CIs) for all strata in the analysis.

**References**

1. World Health Organization. *Global status report on alcohol and health 2018.* Geneva, Switzerland, World Health Organization, 2018,

2. Gu YM, Thijs L, Li Y, Asayama K, Boggia J, Hansen TW, Liu YP, Ohkubo T, Bjorklund-Bodegard K, Jeppesen J, Dolan E, Torp-Pedersen C, Kuznetsova T, Stolarz-Skrzypek K, Tikhonoff V, Malyutina S, Casiglia E, Nikitin Y, Lind L, Sandoya E, Kawecka-Jaszcz K, Imai Y, Mena LJ, Wang J, O'Brien E, Verhamme P, Filipovsky J, Maestre GE, Staessen JA, International Database on Ambulatory blood pressure in relation to Cardiovascular Outcomes (IDACO) Investigators. Outcome-driven thresholds for ambulatory pulse pressure in 9938 participants recruited from 11 populations. *Hypertension.* 2014;63:229-237.

3. Kikuya M, Hansen TW, Thijs L, Björklund-Bodegård K, Kuznetsova T, Ohkubo T, Richart T, Torp-Pedersen C, Lind L, Ibsen H, Imai Y, Staessen JA, on behalf of the International Database on ambulatory blood pressure in relation to Cardiovascular Outcome (IDACO) investigators. Diagnostic thresholds for ambulatory blood pressure monitoring based on 10-year cardiovascular risk. *Circulation.* 2007;115:2145-2152.

4. Levey AS, Stevens LA, Schmid CH, Zhang Y, Castro AF, III, Feldman HI, Kusek JW, Eggers P, Van Lente F, Greene T, Coresh J, for the CKD-EPI (Chronic Kidney Disease Epidemiology Collaboration). A new equation to estimate glomerular filtration rate. *Ann Intern Med.* 2009;150:604-612.

5. Pencina MJ, D'Agostino RB, Sr., D'Agostino RB, Jr., Vasan RS. Evaluating the added predictive ability of a new marker: from area under the ROC curve to reclassification and beyond. *Stat Med.* 2008;27:157-172.

6. Hosmer DW, Jr., Leleshow S. *Applied Logistic Regression*. New York, New York, USA, John Wiley & Sons, 1989, pp 47-56.

**Supplementary Table S1**

**Methods of recruitment and follow-up of the IDCARS cohorts**

| **Study** | **Recruitment** | |  | **Vascular examination (years)** | **Included  in IDCARS** |  | **Follow-up** | |
| --- | --- | --- | --- | --- | --- | --- | --- | --- |
|  | **Sampling  method** | **Starting point  for sample** | **IPR (%)** |  |  |  | **Until  (year)** | **Median in years  (5-95th percentile interval)** |
| Argentina, Buenos Aires | Health check-ups | Out-patient clinic | NA | 2011-2015 | 1428 |  | 2018 | 3.2 (0.4-4.5) |
| Belgium, Noordkempen | Random sample of families | Address lists | 78 | 2005–2015 | 1365 |  | 2018 | 8.8 (3.3-12.3) |
| China, Zhejiang, JingNing | Random sample of families | All villagers invited | 62 | 2003–2008 | 2069 |  | 2012 | 4.0 (3.6-7.6) |
| Czech Republic, Pilsen | Random sample of families | Address list | 82 | 2000–2006 | 206 |  | 2015 | 14.1 (8.5-14.4) |
| Finland, Finrisk | Random sample of community | Population register | 70 | 2007 | 488 |  | 2014 | 6.9 (6.8-6.9) |
| Italy, Padova | Random sample of families | Address lists | 73 | 2006–2008 | 302 |  | 2013 | 6.6 (5.9-7.1) |
| Poland, Gdańsk | Random sample of families | Address list | 90 | 2008–2010 | 297 |  | 2017 | 6.1 (4.8-8.6) |
| Poland, Kraków, Niepolomice | Random sample of families | Address list | 54 | 2001–2008 | 391 |  | 2014 | 12.0 (6.1-12.2) |
| Uruguay, Montevideo | Age-stratified random sample | Members of a health insurance organization | 78 | 2013-2016 | 325 |  | 2016 | 2.3 (1.4-3.1) |

Abbreviation: IPR, initial participation rate. The European Project on Genes in Hypertension included participants recruited in Kraków, Gdańsk, Pilsen and Padova. Participants from Padova were recruited in Mirano in the province of Venice and in Torrebelvicino and Valli del Pasubio in the province of Vicenza. Sample size refers to the number of participants, who underwent an assessment of central hemodynamics at least once. In Uruguay, follow-up was available in a subset of 137 participants. The timing of the vascular examination constituted the baseline.

**Supplementary Table S2**

**Number of endpoints in 5608 participants**

| **Endpoint** |  | **Number of End Points** | | |
| --- | --- | --- | --- | --- |
|  |  | All | Fatal | Nonfatal |
| Cardiovascular endpoint |  | 255 | 64 | 191 |
| Coronary endpoint |  | 109 | 16 | 93 |
| Myocardial infarction |  | 42 | 5 | 37 |
| Coronary revascularization |  | 73 | ... | 73 |
| Sudden death |  | 7 | 7 | ... |
| Heart failure |  | 63 | 13 | 50 |
| Peripheral vascular disease |  | 45 | 2 | 43 |
| Stroke |  | 89 | 26 | 63 |

Median follow-up was 4.1 years (5th-95th percentile interval, 2.2-12.1 years). An ellipsis indicates not applicable. Nonfatal events do not add up, because within each category only the first event was analyzed.

**Supplementary Table S3**

**Cardiovascular and coronary endpoint rates by PPA tertiles in all participants**

| **Endpoints** |  | **Thirds of the PPA distribution** | | |  | **Trend *P*‑Value** |
| --- | --- | --- | --- | --- | --- | --- |
|  |  | **Low** | **Medium** | **High** |  |  |
| PPA limits |  | <1.19 | 1.19-1.36 | ≥1.36 |  |  |
| Number in group |  | 1867 | 1861 | 1880 |  |  |
| Cardiovascular endpoint |  |  |  |  |  |  |
| Number of events (%) |  | 133 (7.1%) | 88 (4.7%) | 34 (1.8%) |  |  |
| Events per 1000 person-years |  | 15.0 (14.2-15.8) | 9.49 (8.81-10.2) | 6.98 (6.39-7.57) |  | <0.001 |
| Coronary endpoint |  |  |  |  |  |  |
| Number of events (%) |  | 55 (2.9%) | 42 (2.3%) | 12 (0.6%) |  |  |
| Events per 1000 person-years |  | 5.26 (4.74-5.78) | 4.37 (3.90-4.84) | 1.53 (1.25-1.81) |  | <0.001 |

Rates expressed in events per 1000 person-years are given with 95% confidence interval.

**Supplementary Table S4**

**Discriminative performance of pulse pressure amplification**

| **Endpoint** |  | **Discovery (n=3945)** |  | **Replication (n=1663)** |  | **All (n=5608)** |
| --- | --- | --- | --- | --- | --- | --- |
| **Cardiovascular** |  |  |  |  |  |  |
| Categorized PPA (<1.3 *vs* ≥1.3) |  |  |  |  |  |  |
| N° events/at risk |  | 150/2230 vs 39/1715 |  | 53/949 vs 13/714 |  | 203/3179 vs 52/2429 |
| Specificity (95% CI) |  | 0.443 (0.427-0.459) |  | 0.435 (0.410-0.459) |  | 0.440 (0.427-0.454) |
| Sensitivity (95% CI) |  | 0.790 (0.712-0.855) |  | 0.750 (0.604-0.834) |  | 0.780 (0.713-0.837) |
| PPV (95% CI) |  | 0.049 (0.040-0.059) |  | 0.038 (0.027-0.052) |  | 0.046 (0.039-0.053) |
| NPV (95% CI) |  | 0.983 (0.976-0.989) |  | 0.983 (0.971-0.991) |  | 0.983 (0.977-0.988) |
| PLR (95% CI) |  | 1.418 (1.295-1.552) |  | 1.327 (1.121-1.571) |  | 1.393 (1.286-1.509) |
| NLR (95% CI) |  | 0.475 (0.343-0.657) |  | 0.575 (0.351-0.942) |  | 0.500 (0.381-0.657) |
| AUC (95% CI) |  | 0.616 (0.581-0.651) |  | 0.592 (0.529-0.655) |  | 0.610 (0.579-0.641) |
| Continuously distributed PPA |  |  |  |  |  |  |
| N° events/at risk |  | 189/3945 |  | 66/1663 |  | 255/5608 |
| AUC (95% CI) |  | 0.644 (0.603-0.685) |  | 0.622 (0.554-0.690) |  | 0.638 (0.603-0.673) |
| **Coronary** |  |  |  |  |  |  |
| Categorized PPA (<1.3 *vs* ≥1.3) |  |  |  |  |  |  |
| N° events/at risk |  | 66/2230 vs 12/1715 |  | 25/949 vs 6/714 |  | 91/3179 vs 18/2429 |
| Specificity (95% CI) |  | 0.439 (0.423-0.454) |  | 0.432 (0.407-0.456) |  | 0.436 (0.423-0.450) |
| Sensitivity (95% CI) |  | 0.836 (0.712-0.922) |  | 0.750 (0.509-0.913) |  | 0.813 (0.707-0.894) |
| PPV (95% CI) |  | 0.021 (0.015-0.027) |  | 0.016 (0.009-0.026) |  | 0.019 (0.015-0.025) |
| NPV (95% CI) |  | 0.995 (0.990-0.998) |  | 0.993 (0.984-0.998) |  | 0.994 (0.990-0.997) |
| PLR (95% CI) |  | 1.490 (1.321-1.680) |  | 1.319 (1.021-1.705) |  | 1.443 (1.292-1.613) |
| NLR (95% CI) |  | 0.373 (0.205-0.679) |  | 0.579 (0.271-1.240) |  | 0.428 (0.266-0.687) |
| AUC (95% CI) |  | 0.637 (0.588-0.687) |  | 0.591 (0.493-0.689) |  | 0.625 (0.580-0.670) |
| Continuously distributed PPA |  |  |  |  |  |  |
| N° events/at risk |  | 78/3945 |  | 31/1663 |  | 109/5608 |
| AUC (95% CI) |  | 0.659 (0.600-0.718) |  | 0.576 (0.475-0.678) |  | 0.637 (0.585-0.688) |

Calculations were performed for the 5‑year risk. PPV is positive predictive value. NPV is negative predictive value. PLR is the positive likelihood ratio (true positive rate/false positive rate. NLR is the negative likelihood ratio (false negative rate/true negative rate). Estimates in this table are unadjusted.


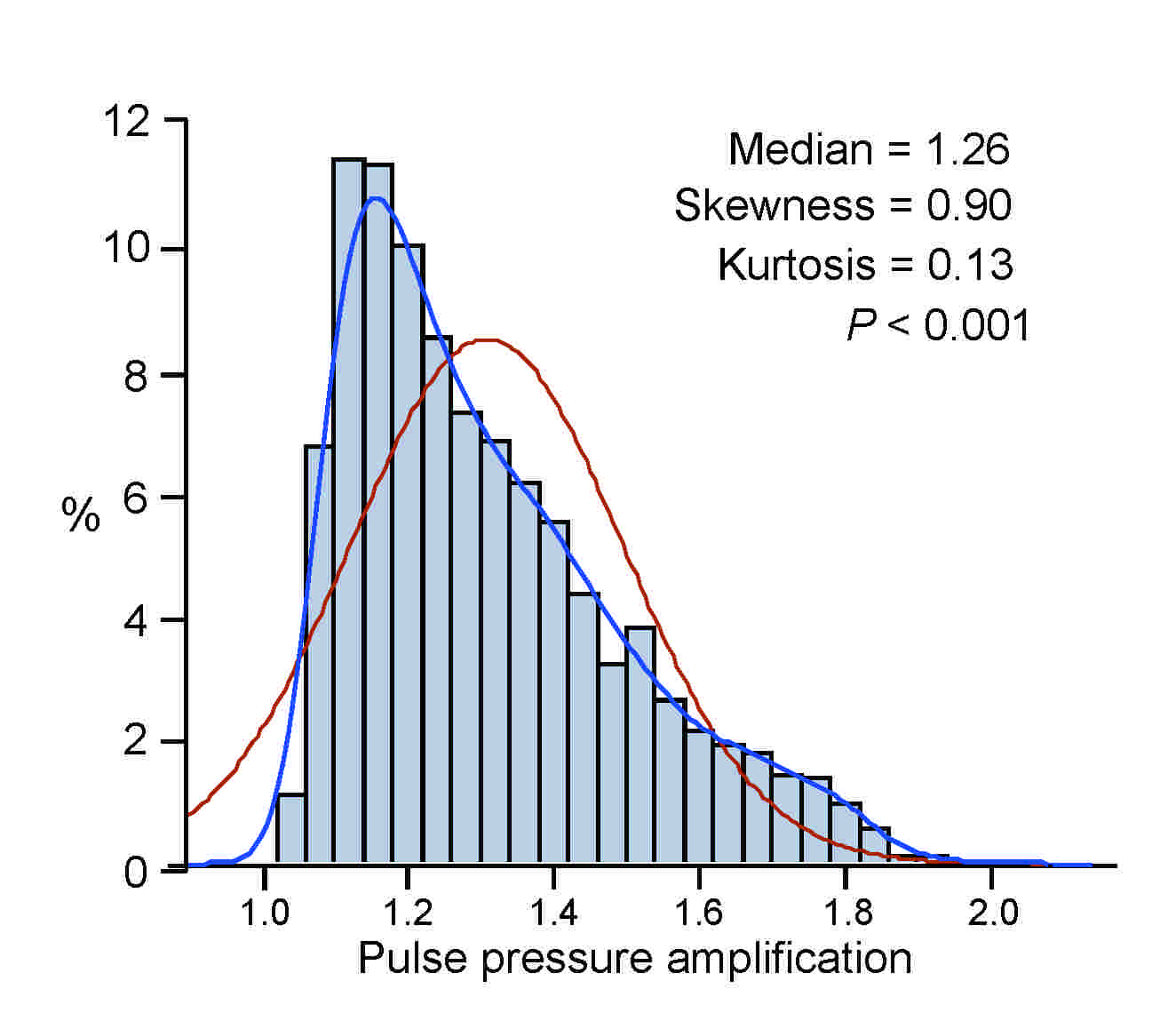


**
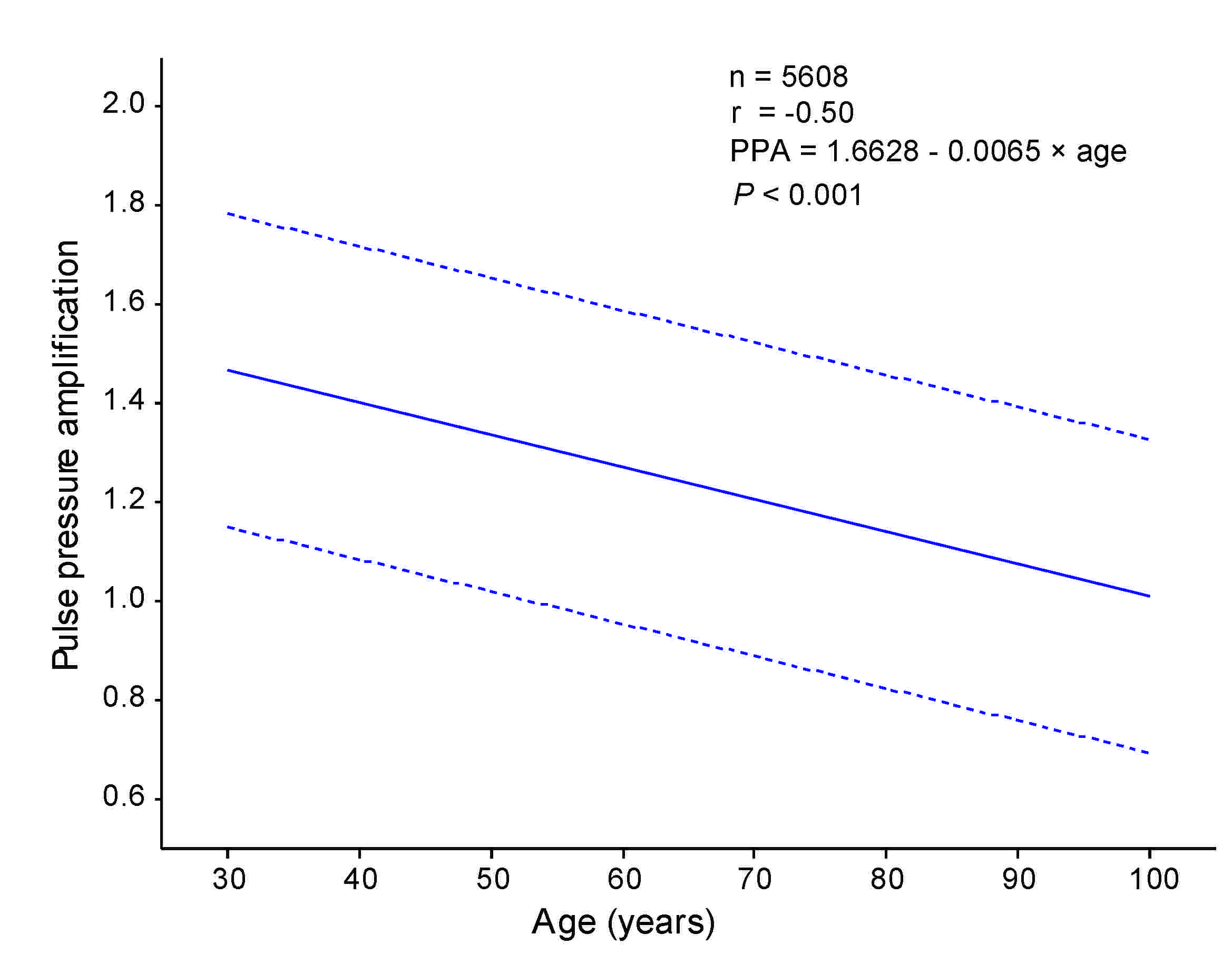
**

**Supplementary Fig. S1**

**Distribution of pulse wave amplification in 5608 IDCARS participants.**

Skewness (S) and kurtosis (K) were computed as the third and fourth moments about the mean divided by the cube of the standard deviation. The black and blue lines represent the normal and kernel density distributions. The *P*-value is for departure of the actually observed distribution from normality according to the Kolmogorov-Smirnov test.

**Supplementary Fig. S2**

**Association between pulse pressure amplification and age in 5608 IDCARS participants**
The regression line is given with the 95% confidence interval for prediction of individual values. The regression equation and *P*‑value are given in the figure panel.
